# BAG2 Interferes with CHIP-Mediated Ubiquitination of HSP72

**DOI:** 10.3390/ijms18010069

**Published:** 2016-12-30

**Authors:** Bianca Schönbühler, Verena Schmitt, Heike Huesmann, Andreas Kern, Martin Gamerdinger, Christian Behl

**Affiliations:** Institute of Pathobiochemistry, University Medical Center of the Johannes Gutenberg University, D-55099 Mainz, Germany; bianca.schoenbuehler@gmail.com (B.S.); schmitt_verena@gmx.net (V.S.); duerk@uni-mainz.de (H.H.); akern@uni-mainz.de (A.K.); martin.gamerdinger@uni-konstanz.de (M.G.)

**Keywords:** aging, BAG2, CHIP, HSP72, proteostasis, ubiquitination

## Abstract

The maintenance of cellular proteostasis is dependent on molecular chaperones and protein degradation pathways. Chaperones facilitate protein folding, maturation, and degradation, and the particular fate of a misfolded protein is determined by the interaction of chaperones with co-chaperones. The co-factor CHIP (C-terminus of HSP70-inteacting protein, STUB1) ubiquitinates chaperone substrates and directs proteins to the cellular degradation systems. The activity of CHIP is regulated by two co-chaperones, BAG2 and HSPBP1, which are potent inhibitors of the E3 ubiquitin ligase activity. Here, we examined the functional correlation of HSP72, CHIP, and BAG2, employing human primary fibroblasts. We showed that HSP72 is a substrate of CHIP and that BAG2 efficiently prevented the ubiquitination of HSP72 in young cells as well as aged cells. Aging is associated with a decline in proteostasis and we observed increased protein levels of CHIP as well as BAG2 in senescent cells. Interestingly, the ubiquitination of HSP72 was strongly reduced during aging, which revealed that BAG2 functionally counteracted the increased levels of CHIP. Interestingly, HSPBP1 protein levels were down-regulated during aging. The data presented here demonstrates that the co-chaperone BAG2 influences HSP72 protein levels and is an important modulator of the ubiquitination activity of CHIP in young as well as aged cells.

## 1. Introduction

The maintenance of intracellular protein homeostasis (proteostasis) is of fundamental importance for the function and viability of cells and includes the controlled coordination of synthesis, folding, assembly, trafficking, and degradation of proteins [1]. The deterioration of proteostasis is associated with several diseases, including neurodegenerative disorders and aging [2,3]. A sensitively regulated network of components is responsible for the maintenance of proteostasis, comprising molecular chaperones, stress-responsive pathways, and protein degradation systems. In healthy conditions, this network shows a basal activity that is rapidly induced upon cellular stress. Molecular chaperones, such as the family of heat shock proteins (HSPs), assist protein folding, preserve metastable protein conformations and refold proteins that have been challenged by denaturation. Moreover, chaperones support the cellular protein degradation pathways and deliver substrates to the ubiquitin proteasome system as well as the autophagic lysosomal system [1,4]. The decision whether a misfolded protein is refolded or directed to degradation, a process termed protein triage, is determined by the activity of a broad network of co-chaperones [5].

CHIP (C-terminus of HSP70-inteacting protein, STUB1) is a highly conserved co-chaperone and is an important factor to direct chaperone activity from folding to degradation [6,7]. The protein is characterized by tandem tetratricopeptide motif repeats at the N-terminus and a U-box domain at the C-terminus that are separated by a charged coiled-coil region [6,8]. CHIP is ubiquitously expressed and its amino acid sequence shows a notable phylogenetic conservation. As an E3 ubiquitin ligase, CHIP is responsible for the covalent ubiquitination of HSP70 client proteins and mediates their degradation. The activity of CHIP converts chaperone/chaperone client complexes in a degradation-competent status and facilitates the removal of misfolded proteins to avoid protein aggregation. In various studies, CHIP has been characterized as a potent factor of the proteostasis network and has been shown to mediate the degradation of several disease-associated factors, such as tau protein, polyglutamine proteins, ataxin-1, SOD1, and α-synuclein [9]. When the proteasome is impaired, CHIP has the potential to shift degradation from the ubiquitin proteasome system to autophagy [10] and, moreover, CHIP is described to directly influence the aging process; CHIP knockout mice show an accelerated aging phenotype [11] and the silencing of CHIP in human primary fibroblasts results in their premature aging [12]. The activity of CHIP is controlled by additional co-chaperones in association with the HSC70/HSP72 complex. Previous studies identified two evolutionary, structurally, and mechanistically distinct HSC70/HSP72 nucleotide exchange factors, BAG2 and HSPBP1, which exhibit at least partial functional redundancy and are both effective inhibitors of the E3 ubiquitin ligase activity of CHIP [13,14,15].

BAG2 is one of the six mammalian proteins (BAG1 to BAG6) that belong to the BCL2-associated athanogene (BAG) protein family, which contain a carboxyl-terminal BAG domain [16]. Some of the BAG proteins share certain domains in addition to the name-giving BAG domain, others are left only with the BAG domain itself [16]. BAG1 is the initially identified anti-apoptotic BCL2-interacting BAG protein and the founding member of the BAG protein family [17]. BAG2 carries only the characteristic BAG domain and was identified as specific inhibitor of the protein CHIP [14]. Both BAG proteins differ in their function and, based on that, their implication in different cellular physiological and pathophysiological functions [16]. Interestingly, there is a high evolutionary conservation of BAG proteins. Homologues are present in *Saccharomyces cerevisiae*, *Drosophila melanogaster*, in amphibians (*Xenopus laevis*), in different non-human mammals (rat and mouse) and plants (e.g., *Arabidopsis thaliana*, *Oryza sativa*). The described evolutionary persistence of BAG proteins suggests a key biological role of BAG domain-carrying proteins in cell physiology [18,19]. The BAG domain has affinity for the ATPase domain of HSP70 family members and facilitates nucleotide exchange at the chaperones. BAG2 has been shown to bind to HSP70/CHIP complexes and to interact with CHIP [14,15]. This remodels chaperone complexes and results in the release of HSC70/HSP72. Moreover, BAG2 is described to directly inhibit the E3 ubiquitin ligase activity of CHIP by disrupting the co-operation between CHIP and its ubiquitin-conjugating E2 enzyme UBCH5A [13]. This modulation of the HSP70/CHIP complex characterizes BAG2 as an important regulator of protein triage decisions and illustrates that the co-chaperone facilitates the pro-folding activity of the cell. The finding that BAG2 is also able to directly bind misfolded proteins and prevent their aggregation [14] strengthens the meaning of the co-chaperone for the cellular protein folding capacity.

HSPBP1 is an additional CHIP inhibitor that binds to HSP70/CHIP complexes and induces conformational changes that interfere with the E3 ubiquitin ligase activity [13]. The finding that two distinct HSC70/HSP72 co-chaperones act as inhibitors of CHIP-mediated ubiquitination illustrates the importance of a fine-tuned regulation of CHIP-mediated protein degradation. Interestingly, both CHIP inhibitors stimulate the maturation of the CFTR at the ER membrane, which reveals their functional redundancy [13,14].

In this study, we investigated the correlation of BAG2, HSP72, and CHIP and show that BAG2 is a potent inhibitor of the CHIP-mediated ubiquitination of HSP72 in young as well as aged primary human fibroblasts. Moreover, we show that BAG2 protein levels are up-regulated in aged cells, which might represent a compensatory mechanism to the age-related accumulation of poly-ubiquitinated proteins, whereas HSPBP1 levels are decreased during aging.

## 2. Results

### 2.1. BAG2 Interferes with CHIP-Mediated Substrate Ubiquitination

**Figure 1:**
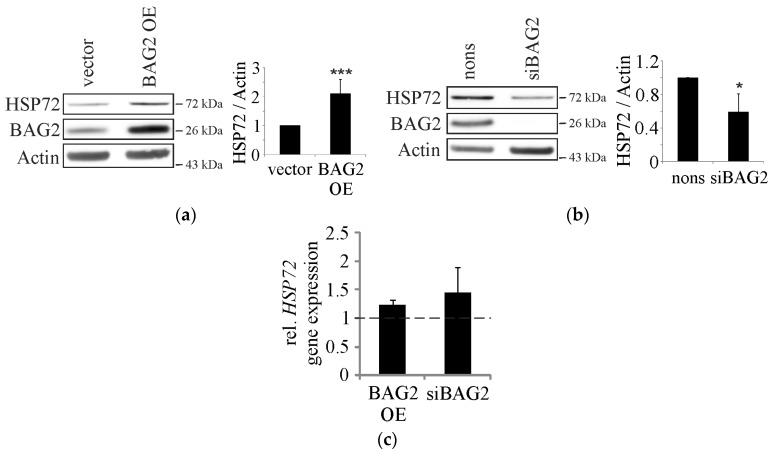
BAG2 modulates HSP72 protein levels. (**a**) Immunoblot analysis of cells that were transfected with the indicated plasmids. Actin served as control for equal loading. Statistics are depicted as mean ± SD. *** *p* < 0.001, *n* = 6; (**b**) Immunoblot analysis of cells that were manipulated with nonsense or BAG2 siRNA. Actin served as loading control. Statistics are depicted as mean ± SD. * *p* < 0.05, *n* = 4; (**c**) Real-time PCR analyses of HSP72 mRNA levels in cells that were manipulated as indicated. Statistics are depicted as mean values (±SD) relative to HSP72 mRNA levels in cells that are treated with empty vector or nonsense siRNA, respectively. *n* = 3.

Previously, BAG2 was characterized as an inhibitor of CHIP that stabilizes chaperone substrates and facilitates the cellular folding activity [14,15]. Here, we observed that protein levels of HSP72, an inducible variant of the HSP70 family, correlate with BAG2 levels (Figure 1a,b); overexpression of BAG2 caused increased HSP72 protein levels and the siRNA-mediated knockdown of BAG2 resulted in reduced levels of the chaperone. Importantly, real-time PCR analyses revealed that mRNA levels of HSP72 were not influenced by alterations in BAG2 levels (Figure 1c), which suggests that BAG2 stabilizes HSP72 on the protein level and might prevent the ubiquitination and subsequent degradation of the chaperone without affecting the sensitively regulated heat shock response.

**Figure 2:**
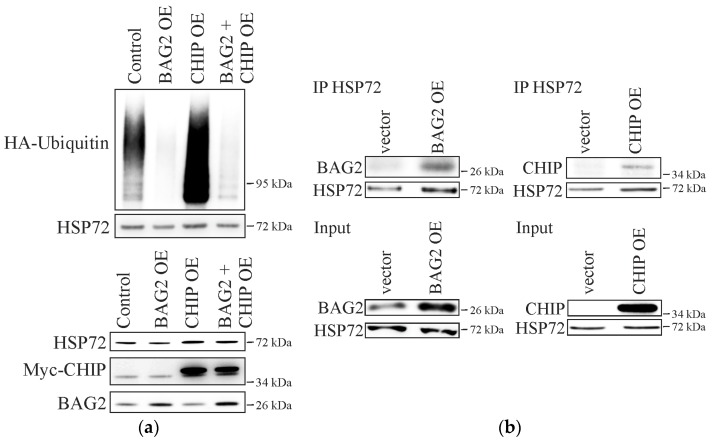
(**a**) Fibroblasts were transiently transfected with HA-tagged ubiquitin and either EGFP as control, or BAG2 or myc-CHIP, or BAG2 and Myc-CHIP. Endogenous HSP72 was immunoprecipitated and the level of ubiquitination was monitored by anti-HA antibody. Purified mouse IgG was used as negative control (not shown). The lower panel shows protein levels in total cell lysates used for Co-IP (Input); (**b**) Cells were transfected with HA-tagged ubiquitin together with either BAG2, Myc-CHIP or EGFP as control. Endogenous HSP72 was immunoprecipitated and BAG2 or myc-CHIP were detected by specific antibodies. The lower panels show protein levels of the analyzed proteins in total cell lysates used for Co-IP (Input). CHIP: C-terminus of HSP70-inteacting protein, STUB1; OE: overexpression; HA: hemagglutinin.

To confirm that HSP72 ubiquitination is dependent on CHIP and that BAG2 influences HSP72 levels by interfering with the activity of the E3 ubiquitin ligase, we immunoprecipitated HSP72 and analyzed the ubiquitination rate of the chaperone by immunoblotting (Figure 2). Note that the detected ubiquitin signal might represent a complex of HSP72 and additional co-chaperones instead of the sole chaperone; however, this approach allows the direct correlation of the total HSP72 ubiquitination rate and the underlying E3 ubiquitin ligase activity. Importantly, increased CHIP levels by overexpression of the protein resulted in an enhanced ubiquitination of HSP72 (Figure 2a). This illustrates that the ubiquitination of the chaperone is dependent on the activity of the E3 ubiquitin ligase and, thus, that HSP72 is indeed a CHIP substrate. However, the overexpression of BAG2 resulted in a strong decrease of the HSP72 ubiquitination rate in comparison to control cells. This inhibition could not be reverted by the simultaneous overexpression of CHIP together with BAG2, which confirms that BAG2 is a potent inhibitor of CHIP and effectively prevents the ubiquitination and subsequent degradation of HSP72.

Importantly, immunoprecipitation studies of HSP72 verified the direct interaction of the chaperone with CHIP as well as BAG2 (Figure 2b), which is a pre-requisite for the CHIP-mediated ubiquitination as well as the inhibitory effect of BAG2 and is consistent with previous data [14,15]. 

These results confirm that BAG2 functions as a potent inhibitor of the E3 ubiquitin ligase activity of CHIP and might modulate the stability of client proteins, such as HSP72, by preventing their ubiquitin-induced degradation.

### 2.2. Cellular Aging Is Associated with Increased CHIP and BAG2 Levels

Aging and multiple age-related disorders are associated with the deterioration of cellular proteostasis, which results in the intracellular accumulation of poly-ubiquitinated proteins and protein aggregates [3]. Aged cells are characterized by alterations in chaperone levels and changed activities of the protein degradation pathways. In human primary fibroblasts, CHIP is described to be up-regulated during aging and the silencing of the co-chaperone in young cells results in their premature senescence [12,20]. Here, we employed human primary fibroblasts as a replicative senescence model system and analyzed potential alterations in BAG2 levels and function during cellular aging.

**Figure 3:**
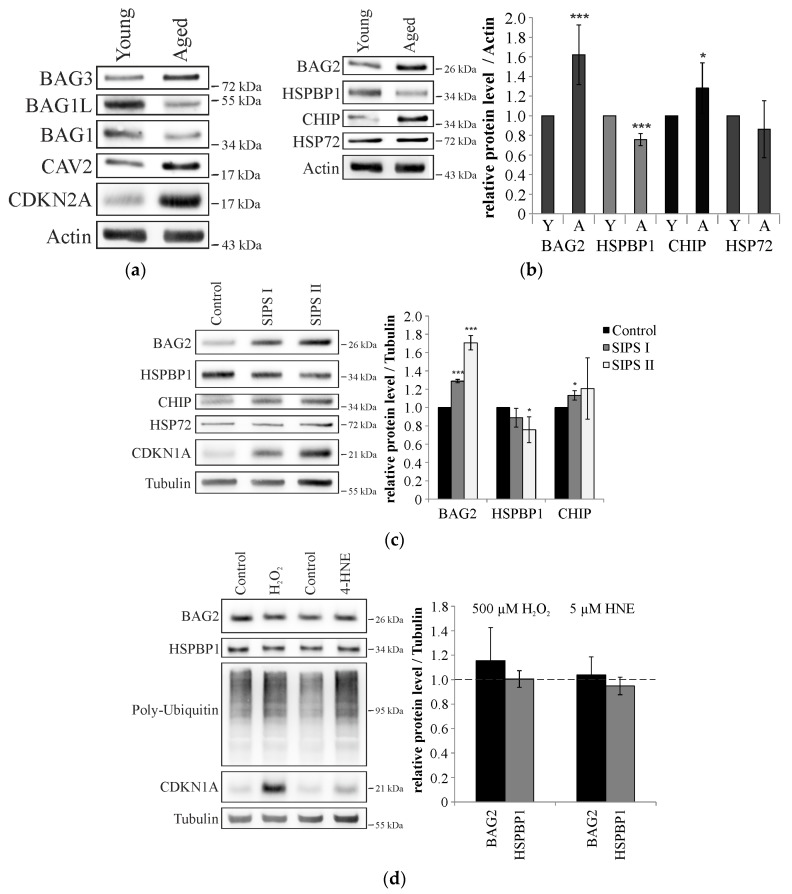
(**a**) Immunoblot analysis of aging marker proteins in young (PDL 20) and aged (PDL + 50) fibroblasts. Actin served as control of equal loading. One representative blot is shown. *n* = 3; (**b**) Immunoblot analysis of BAG2, HSPBP1, CHIP, and HSP72 in young and aged fibroblasts. Actin served as loading control. Statistics are depicted as mean ± SD. * *p* < 0.05, *** *p* < 0.001, *n* = 4; (**c**) Immunoblot analysis of indicated proteins in young fibroblasts, that were treated repeatedly with 200 µM (SIPS I) and 500 µM (SIPS II) hydrogen peroxide (H_2_O_2_) to induce premature senescence (SIPS). Tubulin served as loading control. Statistics are depicted as mean ± SD. * *p* < 0.05, *** *p* < 0.001, *n* = 3; (**d**) Immunoblot analysis of BAG2 and HSPBP1 levels in fibroblasts that were treated with H_2_O_2_ or 4-hydroxy-nonenal (4-HNE). Poly-Ubiquitin and CDKN1A indicate proteostatic stress. Tubulin served as loading control. Statistics are depicted as mean ± SD. *n* = 3.

The human fibroblasts were cultivated until their culture showed defined signs of aging [21], such as an arrested cell cycle, an altered morphology, and alterations in aging marker proteins, including the reciprocal regulation of BAG1 and BAG3 protein levels [22] as well as increased levels of CAV2 and CDKN2A/p16 [23] (Figure 3a). Employing these senescent cells, we analyzed total protein levels of BAG2, CHIP, HSP72, and HSPBP1 (Figure 3b). Importantly, we confirmed that protein levels of CHIP are up-regulated in aged fibroblasts and demonstrate that also BAG2 levels are increased during cellular aging. Thus, CHIP and BAG2 levels follow an equal adaptation to the aged cellular environment. Furthermore, we observed that protein levels of the stress-inducible chaperone HSP72 were unaltered in senescent cells and that HSPBP1 protein levels were down-regulated.

To confirm that the observed alterations are indeed related to cellular aging, we repetitively treated young fibroblast cultures with sub-lethal concentrations of hydrogen peroxide to gain stress-induced premature senescent cells [24]. Premature senescence is characterized by distinct aging phenotypes, including cell cycle arrest, altered morphology, and the up-regulation of aging marker proteins, such as CAV2 and CDKN1A/p21 (Figure 3c). Importantly, the analysis of BAG2, CHIP, and HSPBP1 protein levels in these premature senescent cells showed an equal regulation as we observed during replicative senescence; BAG2 and CHIP protein levels were up-regulated, whereas HSPBP1 levels were reduced compared to control cells. Acute stress conditions induced by the treatment of fibroblasts with high concentrations of hydrogen peroxide or 4-hydroxy-2-nonenal led to a deterioration of proteostasis and resulted in the accumulation of poly-ubiquitinated proteins, but left BAG2 and HSPBP1 protein levels unaltered (Figure 3d). Thus, the reciprocal regulation of both co-chaperones observed in aged fibroblasts seems to be directly related to the aging process.

Since BAG2 and HSPBP1 are at least partially redundant in their functions, we analyzed whether BAG2 itself has the potential to influence HSPBP1 protein levels (Figure 3e). Interestingly, endogenous HSPBP1 protein levels were unaffected by overexpression as well as siRNA-mediated knockdown of BAG2. Obviously, the age-related reciprocal regulations of BAG2 and HSPBP1 appear specifically during cellular aging and the decreased HSPBP1 protein levels are not a direct consequence of increased BAG2 protein levels.

### 2.3. BAG2 Inhibits CHIP-Mediated HSP72 Ubiquitination in Aged Cells

**Figure 4:**
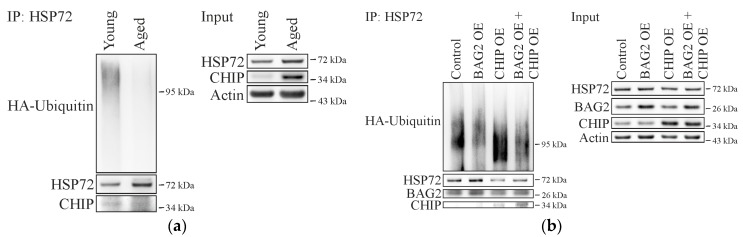
(**a**) Co-IP analysis of young and aged fibroblasts that were transfected with HA-tagged ubiquitin and Flag-tagged HSP72. The accumulation of poly-ubiquitinated proteins was induced by MG132 treatment (4 µM, 4 h) prior to cell lysis. The levels of HSP72 ubiquitination were visualized by immunoblot analysis (left panel). The input fraction is shown on the right panel; (**b**) Aged fibroblasts were transiently transfected with HA-tagged ubiquitin and FLAG-tagged HSP72 and either EGFP (control), BAG2, CHIP or with BAG2 and CHIP. The accumulation of poly-ubiquitinated proteins was induced by treatment with MG132 (4 µM, 4 h) prior to cell lysis. HSP72 was immunoprecipitated and levels of ubiquitination proteins were verified by immunoblot analysis (left panel). Purified mouse IgG was used as control (not shown). The right panel shows relative protein levels in cell lysates used for Co-IP (Input).

Since protein levels of CHIP as well as BAG2 were increased in senescent fibroblasts, we were interested in the functional consequence of this regulation and compared the total ubiquitination status of HSP72 in aged and young fibroblasts by immunoprecipitation of the chaperone. Interestingly, the level of ubiquitinated HSP72 was strongly decreased in aged compared to young cells (Figure 4a), highlighting that the ubiquitination activity of CHIP declines during aging. Due to this observation, we analyzed the functional capacity of CHIP and BAG2 in aged cells by overexpression of both proteins. Importantly, increasing CHIP levels caused an enhanced ubiquitination rate of HSP72, while overexpressing BAG2 decreased the ubiquitination of the chaperone (Figure 4b). Moreover, the simultaneous overexpression of CHIP and BAG2 resulted in a reduced level of HSP72 ubiquitination. Thus, cellular aging does not affect the distinct function of BAG2 and CHIP, but the age-related up-regulation of BAG2 effectively counteracts the activity of the E3 ubiquitin ligase, which results in a decline in CHIP-mediated ubiquitination of HSP72.

## 3. Discussion

Cellular proteostasis is continuously challenged by different forms of stress. Cells have the capacity to adapt the proteostasis network to counteract these stress conditions, but particularly during aging, the adjustments are not sufficient to prevent a global decline in proteome integrity [2,4]. Thus, aging and several age-related neurodegenerative diseases are accompanied by the accumulation of misfolded, poly-ubiquitinated proteins and protein aggregates.

In this study, we analyzed the functional correlation of the proteostasis network factors HSP72, BAG2, and CHIP. The co-chaperone BAG2 is a nucleotide exchange factor for HSC70/HSP70 and a potent inhibitor of CHIP, which modulates the fate of chaperone substrates and the decision between folding and degradation [14,15]. By the inhibition of the E3 ubiquitin ligase activity of CHIP, BAG2 facilitates the cellular pro-folding capacity of chaperones. Here, we show that the fate of the stress inducible HSP70 family member HSP72 is modulated by BAG2 very likely without influencing the heat shock response. We demonstrate that HSP72 is a CHIP substrate and show that BAG2 efficiently prevents the ubiquitination of the chaperone. Indeed, BAG2 levels correspond with protein levels of HSP72; increased BAG2 levels stabilized HSP72 by inhibition of its CHIP-mediated ubiquitination, whereas HSP72 ubiquitination is enhanced when BAG2 levels are decreased. Thus, BAG2 interferes with the activity of CHIP and prevents the ubiquitination of CHIP substrates. This finding is consistent with a previous study that analyzed the impact of BAG2 on CHIP employing recombinant proteins and demonstrated that BAG2 efficiently prevents the co-operation of CHIP with the ubiquitin-conjugating E2 enzyme UBCH5B [14]. CHIP has been widely studied as a central factor of proteostasis that counteracts pathological conditions, such as neurodegeneration, by inducing the effective degradation of misfolded and aggregation-prone proteins [9]. The E3 ubiquitin ligase is responsible for the ubiquitination of an extensive portfolio of HSC70/HSP70-bound substrates, which overall serves cellular protein quality control. Importantly, it has been shown that CHIP preferentially ubiquitinates chaperone-bound substrates, but when these are depleted the co-chaperone also tags chaperones and delivers them for degradation to the proteasome [25].

Interestingly, CHIP has been described as a direct modulator of aging [11,12], which highlights the importance of the co-chaperone for the maintenance of cellular proteostasis. We confirmed the age-associated up-regulation of CHIP in aged human fibroblasts and showed that also protein levels of its inhibitor, BAG2, are increased during aging. This synchronous regulation provoked the analysis of the functional consequence and the actual ubiquitination activity of CHIP in senescent cells. Importantly, overexpression of CHIP in aged fibroblasts resulted in an enhanced HSP72 ubiquitination. This demonstrates that the activity of CHIP is not disturbed in the aged environment, but, excitingly, when we compared the ubiquitination of HSP72 in young and aged cells, we observed that the total ubiquitination rate of the chaperone was strongly decreased. Indeed, BAG2 retained its potential to inhibit CHIP in aged cells and increased levels of BAG2 effectively prevented the CHIP-mediated ubiquitination of HSP72. Thus, although CHIP is up-regulated in aged cells its activity is reduced by BAG2. This illustrates that the pro-folding activity is increased in aged cells. Aging is characterized by a decline in the activity of the protein degradation systems, which results in the accumulation of misfolded and poly-ubiquitinated proteins [26]. In this environment, it seems rational to increase the protein folding capacity of the cell and to decrease the total ubiquitination activity. Interestingly, within the family of BAG proteins, besides BAG2 also BAG1 has been implicated in CHIP-mediated protein degradation [27]. The co-chaperone cooperates with CHIP and facilitates the transfer of ubiquitinated proteins to the proteasome and, importantly, BAG1 protein levels are decreased in senescent fibroblasts [22]. This regulation of BAG1 reduces the activity of the ubiquitin proteasome system and enhances the accumulation of poly-ubiquitinated proteins within the cell. The up-regulation of BAG2 prevents protein ubiquitination by CHIP and increases the folding capacity, which will be of benefit for the aged cell.

In contrast to BAG2, protein levels of the CHIP inhibitor HSPBP1 were down-regulated during replicative senescence. This reciprocal adjustment of BAG2 and HSPBP1 occurs specifically during cellular aging and not as a compensatory mechanism or a general response to stress conditions. The existence of two evolutionary, structurally, and mechanistically distinct co-chaperones with partial functional redundancy demonstrates the importance of a stringent regulation of CHIP, which will coordinate protein triage decisions and will facilitate the maintenance of proteostasis in the aged environment.

In conclusion, our results demonstrate that the co-chaperone BAG2 influences HSP72 protein levels and is an important modulator of the activity of CHIP. BAG2 retains this CHIP-modulatory potential in the course of cellular aging. Further elucidation of the complex regulation of the E3 ubiquitin ligase by BAG2 and HSPBP1 will contribute to a better understanding of decisions supporting protein folding or degradation and the regulation of these mechanisms in the course of cellular aging.

## 4. Materials and Methods

### 4.1. Cell Culture

Human primary fibroblasts (IMR90, Coriell Institute of Medical Research, Camden, NJ, USA) were cultivated in DMEM supplemented with 1 mM sodium pyruvate, 1× non-essential amino acids, 10% FCS and 1× antibiotic/antimitotic solution at 37 °C in a 5% CO_2_-humidified atmosphere. Fibroblasts were cultured at sub-confluency and cell numbers were calculated with every passage to follow population doubling levels (PDL) exactly as described in [22,23]. At PDL 50, aging markers were analyzed and the fibroblast cultures were defined as aged. To induce premature senescence, young fibroblasts were treated with indicated concentrations of hydrogen peroxide for 2 h. After 4 days, a second treatment followed and 3 days later the cells were analyzed for aging markers.

### 4.2. Plasmids, siRNAs and Transfection

For the generation of expression plasmids of human BAG2 (pBAG2-N1) and CHIP (pCHIP-N1), cDNAs of the respective genes were fused into pEGFP-N1 (Clontech, Mountain View, CA, USA) linearized with HindIII and BsrGI. Expression plasmids for FLAG-tagged human HSP72 (FLAG-HSP70) were generated by fusing the PCR amplificate into pFLAG-BAG3-N1 [18], linearized with BamHI and NotI. For the generation of each plasmid, the fusing reaction was accomplished using the In-Fusion Advantage PCR Cloning Kit (Clontech) according to manufacturer’s protocol. The pRK5-HA-ubiquitin expression plasmid was obtained from Addgene. For siRNA-mediated knockdown, cells were transfected with 20 µg of siRNA (Eurofins MWG Operon, Ebersberg, Germany) by electroporation, exactly as described in [18]. For each experiment, a control was transfected with the same amount of an empty vector, an EGFP encoding plasmid, or a nonsense siRNA. Sequences of all siRNAs used in this study are listed in Supplementary Table S1.

### 4.3. Immunoblotting

Immunoblotting was accomplished as previously described [22]. In short, equal amounts of total protein were subjected to SDS-PAGE, employing hand-cast 10% Bis-Tris gels or precast 4%–12% NuPAGE Bis-Tris gels (Invitrogen, Karlsruhe, Germany). All antibodies used in this study are listed in Supplementary Table S2.

### 4.4. Real-Time PCR

Real-Time PCR analyses were conducted as previously described [28]. Briefly, RNA was extracted from cells employing the NucleoSpin RNA II Kit (Macherey-Nagel, Weilmünster, Germany) according to the manufacturer’s instructions. Reverse transcription was performed with 1 µg of total RNA using the Omniscript RT Kit (Qiagen, Hilden, Germany). Quantitative real-time PCR was performed in the iCycler real-time thermocycler (Biorad, Dreieich, Germany). Relative gene expression ratios were determined with the REST software [29].

### 4.5. Co-Immunoprecipitation and Ubiquitination Assay

Cells were transiently transfected with the mammalian expression plasmid pRK5-HA-ubiquitin and with indicated additional plasmids. To enhance the ubiquitin signal in senescent fibroblasts, poly-ubiquitinated proteins were accumulated by the treatment with MG132 (4 µM, 4 h) prior to cell lysis. Ubiquitin-conjugated constituents of the HSP72 complex were detected using an anti-HA antibody. For co-immunoprecipitation, cells were lysed for 15 min on ice with IP-buffer (50 mM Tris-HCl pH 7.5, 150 mM NaCl, 2 mM EDTA, 1 mM EGTA, 0.5% NP40, 50 mM *N*-Ethylmaleimid). Lysates were centrifuged (10 min, 1000× *g*, 4 °C) and the supernatant was used for immunoprecipitation. Equal amounts of protein were supplemented with 1 µg of antibody and incubated for 1 h at 4 °C. Subsequently, protein G sepharose beads (GE Healthcare, Freiburg, Germany) were added and samples were incubated at 4 °C for 1 h. Immunoprecipitated proteins were subjected to SDS-PAGE and visualized by immunoblotting.

### 4.6. Statistical Analysis

All results are expressed as mean ± standard deviation (SD). Statistical significance was obtained by Student’s *t*-test, employing the SIGMA STAT software (SPSS Science, Erkrath, Germany).

## Supplementary Materials

Supplementary materials can be found at www.mdpi.com/1422-0067/18/1/69/s1.

## Abbreviations

BAG2	BCL2-associated athanogene 2
CFTR	Cystic fibrosis transmembrane conductance regulator
CHIP	C-terminus of HSP70-interacting protein
HSP	Heat shock protein
HSPBP1	HSP70 binding protein 1

## References

[1] Hartl F.U., Bracher A., Hayer-Hartl M. (2011). Molecular chaperones in protein folding and proteostasis. Nature.

[2] Douglas P.M., Dillin A. (2010). Protein homeostasis and aging in neurodegeneration. J. Cell Biol..

[3] Lopez-Otin C., Blasco M.A., Partridge L., Serrano M., Kroemer G. (2013). The hallmarks of aging. Cell.

[4] Hipp M.S., Park S.H., Hartl F.U. (2014). Proteostasis impairment in protein-misfolding and -aggregation diseases. Trends Cell Biol..

[5] Hohfeld J., Cyr D.M., Patterson C. (2001). From the cradle to the grave: Molecular chaperones that may choose between folding and degradation. EMBO Rep..

[6] Ballinger C.A., Connell P., Wu Y., Hu Z., Thompson L.J., Yin L.Y., Patterson C. (1999). Identification of CHIP, a novel tetratricopeptide repeat-containing protein that interacts with heat shock proteins and negatively regulates chaperone functions. Mol. Cell. Biol..

[7] Murata S., Minami Y., Minami M., Chiba T., Tanaka K. (2001). CHIP is a chaperone-dependent E3 ligase that ubiquitylates unfolded protein. EMBO Rep..

[8] Connell P., Ballinger C.A., Jiang J., Wu Y., Thompson L.J., Hohfeld J., Patterson C. (2001). The co-chaperone CHIP regulates protein triage decisions mediated by heat-shock proteins. Nat. Cell Biol..

[9] Paul I., Ghosh M.K. (2015). A chipotle in physiology and disease. Int. J. Biochem. Cell Biol..

[10] Sha Y., Pandit L., Zeng S., Eissa N.T. (2009). A critical role for CHIP in the aggresome pathway. Mol. Cell. Biol..

[11] Min J.N., Whaley R.A., Sharpless N.E., Lockyer P., Portbury A.L., Patterson C. (2008). CHIP deficiency decreases longevity, with accelerated aging phenotypes accompanied by altered protein quality control. Mol. Cell. Biol..

[12] Sisoula C., Gonos E.S. (2011). CHIP E3 ligase regulates mammalian senescence by modulating the levels of oxidized proteins. Mech. Ageing Dev..

[13] Alberti S., Bohse K., Arndt V., Schmitz A., Hohfeld J. (2004). The cochaperone HSPBP1 inhibits the CHIP ubiquitin ligase and stimulates the maturation of the cystic fibrosis transmembrane conductance regulator. Mol. Biol. Cell.

[14] Arndt V., Daniel C., Nastainczyk W., Alberti S., Hohfeld J. (2005). Bag-2 acts as an inhibitor of the chaperone-associated ubiquitin ligase CHIP. Mol. Biol. Cell.

[15] Dai Q., Qian S.B., Li H.H., McDonough H., Borchers C., Huang D., Takayama S., Younger J.M., Ren H.Y., Cyr D.M. (2005). Regulation of the cytoplasmic quality control protein degradation pathway by Bag-2. J. Biol. Chem..

[16] Behl C. (2016). Breaking bag: The co-chaperone Bag-3 in health and disease. Trends Pharmacol. Sci..

[17] Takayama S., Sato T., Krajewski S., Kochel K., Irie S., Millan J.A., Reed J.C. (1995). Cloning and functional analysis of Bag-1: A novel BCL-2-binding protein with anti-cell death activity. Cell.

[18] Doukhanina E.V., Chen S., van der Zalm E., Godzik A., Reed J., Dickman M.B. (2006). Identification and functional characterization of the bag protein family in arabidopsis thaliana. J. Biol. Chem..

[19] Takayama S., Reed J.C. (2001). Molecular chaperone targeting and regulation by bag family proteins. Nat. Cell Biol..

[20] Sisoula C., Trachana V., Patterson C., Gonos E.S. (2011). CHIP-dependent p53 regulation occurs specifically during cellular senescence. Free Radic. Biol. Med..

[21] Campisi J., d’Adda di Fagagna F. (2007). Cellular senescence: When bad things happen to good cells. Nat. Rev. Mol. Cell Biol..

[22] Gamerdinger M., Hajieva P., Kaya A.M., Wolfrum U., Hartl F.U., Behl C. (2009). Protein quality control during aging involves recruitment of the macroautophagy pathway by Bag-3. EMBO J..

[23] Kern A., Roempp B., Prager K., Walter J., Behl C. (2006). Down-regulation of endogenous amyloid precursor protein processing due to cellular aging. J. Biol. Chem..

[24] Toussaint O., Medrano E.E., von Zglinicki T. (2000). Cellular and molecular mechanisms of stress-induced premature senescence (SIPS) of human diploid fibroblasts and melanocytes. Exp. Gerontol..

[25] Qian S.B., McDonough H., Boellmann F., Cyr D.M., Patterson C. (2006). CHIP-mediated stress recovery by sequential ubiquitination of substrates and HSP70. Nature.

[26] Morawe T., Hiebel C., Kern A., Behl C. (2012). Protein homeostasis, aging and Alzheimer’s disease. Mol. Neurobiol..

[27] Luders J., Demand J., Hohfeld J. (2000). The ubiquitin-related Bag-1 provides a link between the molecular chaperones Hsc70/HSP70 and the proteasome. J. Biol. Chem..

[28] Spang N., Feldmann A., Huesmann H., Bekbulat F., Schmitt V., Hiebel C., Koziollek-Drechsler I., Clement A.M., Moosmann B., Jung J. (2014). RAB3GAP1 and RAB3GAP2 modulate basal and rapamycin-induced autophagy. Autophagy.

[29] Pfaffl M.W., Horgan G.W., Dempfle L. (2002). Relative expression software tool (REST) for group-wise comparison and statistical analysis of relative expression results in real-time PCR. Nucleic Acids Res..

