# Supplementary material for: BAG2 Interferes with CHIP-Mediated Ubiquitination of HSP72
Source: Int J Mol Sci. 2016 Dec 30;18(1):69. doi: 10.3390/ijms18010069 (PMC5297704; doi:10.3390/ijms18010069)
Supplement: Supplementary file 1 [file ijms-18-00069-s001.pdf]

Supplementary Materials: BAG2 Interferes with CHIP-Mediated Ubiquitination of HSP72

Bianca Schönbühler, Verena Schmitt, Heike Huesmann, Andreas Kern, Martin Gamerdinger and Christian Behl

Table S1. Small interfering RNA (siRNA) sequences.

Name	Sequence 5′–3′
BAG2 siRNA	AGAUUCCUGUGUCUCAUAA
HSPBP1 siRNA	UCAGCAUUCCUGCUGCAGA
Nonsense siRNA	AUUCUCCGAACGUGUCACG

Table S2. Antibodies.

Antibodies	Sources and Suppliers
polyclonal anti-STUB1 (CHIP)	ab2917; Abcam (Cambridge, UK)
monoclonal anti-Hsp70/HSP72	ADI-SPA-810; Stressgen (via Enzo Life Sciences, Lörrach, Germany)
polyclonal anti-BAG2	2610-1; Epitomics (Burlingame, CA, USA)
polyclonal anti-HSPBP1	DB090; Delta Biolabs (Gilroy, CA, USA)
polyclonal anti-BAG domain	BAG1M aa151-263 (provided by Ulrich Hartl)
polyclonal anti-BAG3	10599-1-AP; Proteintech Group (Manchester, UK)
polyclonal anti-Ubiquitin	Z0458; Dako (Glostrup, Denmark)
monoclonal anti-p21	554228;BD Biosciences (San Jose, CA, USA)
monoclonal anti-Caveolin 2	610685;BD Biosciences(San Jose, CA, USA)
monoclonal anti-Tubulin	T9026; Sigma (Munich, Germany)
monoclonal anti-Gapdh	ab9482; Abcam (Cambridge, UK)
polyclonal anti-Actin	A5060; Sigma (Munich, Germany)
polyclonal anti HA-Tag	H6908; Sigma (Munich, Germany)
monoclonal anti-c-Myc	631206; Clontech (Mountain View, CA, USA)
